# Phenolic content and antioxidant property of the bark extracts of *Ziziphus mucronata *Willd. subsp. *mucronata *Willd

**DOI:** 10.1186/1472-6882-11-130

**Published:** 2011-12-16

**Authors:** Olufunmiso O Olajuyigbe, Anthony J Afolayan

**Affiliations:** 1 Phytomedicine Research Centre, Department of Botany, University of Fort Hare, Alice, 5700, South Africa

## Abstract

**Background:**

Several plants traditionally used in treatment of a variety of infections in South Africa are reported in ethnobotanical surveys. Many of these plants including *Ziziphus mucronata *subsp. *mucronata *lack scientific reports to support their medicinal importance.

**Methods:**

The antioxidant activities and phenolic contents of the acetone, ethanol and aqueous extracts of the stems of *Z. mucronata *subsp. *mucronata *were evaluated using *in vitro *standard methods. The total phenol, total flavonoids and proanthocyanidin content were determined spectrophotometrically. Quercetin, Tannic acid and catechin equivalents were used for these parameters. The antioxidant activities of the stem bark extracts of this plant were determined by ABTS, DPPH, and ferrous reducing antioxidant property (FRAP) methods.

**Results:**

The quantity of the phenolic compounds, flavonoids and proanthocyanidins detected differ significantly in the various extracts. The phenolics were significantly higher than the flavonoids and proanthocyanidin contents in all the extracts investigated. The ferric reducing ability and the radical scavenging activities of the extracts were very high and dose-dependent. The ethanol extract had the highest antioxidant activity, followed by the acetone extract while the aqueous extract was the least active. Reacting with ABTS, the 50% inhibitory concentrations (IC_50_) were (0.0429 ± 0.04 mg/ml) for aqueous, (0.0317 ± 0.04 mg/ml) for acetone and (0.0306 ± 0.04 mg/ml) for ethanol extracts while they inhibited DPPH radical with 50% inhibitory concentration (IC_50_) values of 0.0646 ± 0.02 mg/ml (aqueous), 0.0482 ± 0.02 mg/ml (acetone) and 0.0422 ± 0.03 mg/ml (ethanol).

**Conclusions:**

A correlation between the antioxidant activity and the total phenolic contents of the extracts indicated that phenolic compounds were the dominant contributors to the antioxidant activity of the plant. This study, therefore, demonstrated that *Z. mucronata *subsp*. mucronata *has strong antioxidant property and free radical scavenging capability.

## Background

Free radicals and reactive oxygen species (ROS) such as superoxide, hydroxyl and peroxyl radicals are normal by-products of aerobic metabolism produced *in vivo *during oxidation [1]. These ROS are generated in the mitochondria and microsome organelles under normal physiological conditions. They can also be produced externally by exposure to radiation, toxic chemicals, cigarette smoking and alcohol consumption, and by eating oxidized polyunsaturated fats. Overproduction of ROS can result in oxidative damage to various biomolecules including lipids, proteins, DNA and cell membranes [2]. They also lead to the development of a variety of diseases such as coronary heart diseases, cancer, diabetes, hypertension and neurodegeneration [3,4]. While compounds capable of scavenging free radicals possess great potential in ameliorating these diseases [5,6], most of the ROS are scavenged by endogenous defense enzymes such as catalase, superoxide dismutase and peroxidase-glutathione system [7]. However, the activities of these endogenous defense systems may not be sufficient to mop up the free radicals.

Commonly used synthetic antioxidants include butylated hydroxyanisole (BHA), butylated hydroxytoluene (BHT), propylgallate (PG) and tertbutylhydroxyltoluene (TBHQ) [8]. Though important, they are known to constitute potential health risks and toxic effects [9]. Their applications are, therefore, strongly restricted. Hence, the need to search, develop and utilize more effective antioxidant from natural origin [10]. The medicinal properties of many plants have been attributed to the antioxidant properties of their constituents [11]. Some epidemiological studies also showed that the consumption of some plants can protect humans against oxidative damage by quenching free radicals and ROS [12,13]. Today, there are overwhelming interests in finding naturally occurring antioxidants for use in foods and in medicinal materials to replace synthetic antioxidants [14]. Interests in plant materials that are rich in phenols are increasing among scientists, food manufacturers and consumers because these materials retard oxidative degradation of lipids and improve the quality and nutritional value of food. People tend to eat functional foods with specific health benefits. The roles of natural antioxidants, mainly phenolic compounds, believed to have more antioxidant activity than vitamins C, E and β-carotene [15] is rapidly gaining attention.

*Ziziphus mucronata *Willd. subsp*. mucronata *Willd*.*, also known as buffalo thorn, is a small to medium-sized tree, with a spreading canopy. It is distributed throughout the summer rainfall areas of sub-Saharan Africa, extending from South Africa northwards to Ethiopia and Arabia. Its bark and roots are used medicinally for the treatment of various ailments, including rheumatism, gastrointestinal complaints and snake bites [16]. Warm bark infusions are used for body pains, expectorants in cough, respiratory infections and chest problems. The root infusions are used for treating gonorrhea, diarrhoea and dysentery. Decoctions of roots and leaves are applied externally to boils, sores and glandular swellings not to promote healing but for pain relief [17,18]. While several species of the genus *Ziziphus *have been investigated, there is a dearth of information on the phytochemical property and antioxidant activities of this plant. Hence, in line with the current trend of finding naturally occurring antioxidants, this study was designed to investigate the phenolic contents and antioxidant potential of different extracts of *Ziziphus mucronata *subsp. *mucronata *to justify its ethnomedicinal importance.

## Methods

### Collection of plant material

The bark materials of *Z. mucronata *subsp*. mucronata *were collected from the plant growing within the University of Fort Hare campus in Alice, South Africa. The plant was authenticated in the Department of Botany by Prof. Grierson and a voucher specimen (OLAJ/2010/ZM/01) was prepared and deposited in the Griffin's Herbarium of the University.

The bark samples were air-dried at room temperature and pulverized using a milling machine. Portions of about 100 g each of the pulverized samples were extracted separately with 500 ml of each of the solvents, acetone, ethanol and water, for 48 h. The extracts were filtered through Whatman No. 1 filter paper and evaporated to dryness under reduced pressure at 40°C using a rotary evaporator. The filtrate of aqueous extract obtained was quickly frozen at -40°C and dried for 48 h using a freeze dryer (Savant Refrigerated vapor Trap, RV T41404, USA). The extraction yielded 5.3 g acetone extract, 14.2 g ethanol extract and 15.8 g water extract. The extracts were redissolved in their respective solvents to the required concentrations for the bioassay analysis.

### Chemicals and reagents used

2,2'-azinobis-3-ethylbenzothiazoline-6-sulfonic acid (ABTS) diammonium salt, 1,1-diphenyl-2-picrylhydrazyl (DPPH), butylated hydroxytoluene (BHT), gallic acid, rutin, ascorbic acid (VC), quercetin and FeCl_3_, were purchased from Sigma Chemical Co. (St. Louis, MO, USA); vanillin was from BDH Chemicals Ltd. (Poole, England) and Folin-Ciocalteu phenol reagent and sodium carbonate were from Merck Chemical Supplies (Darmstadt, Germany). All other chemicals used, including the solvents, were of analytical grade.

### Determination of total flavonoids

Total flavonoids were estimated using the method of Ordonez et al. [19]. Here, 0.5 ml of 2% AlCl_3 _ethanol solution was added to 0.5 ml of extract and allowed to stand for 60 min at room temperature before the absorbance was measured at 420 nm. The extract was evaluated at a final concentration of 1 mg/ml. Total flavonoids content was calculated as quercetin equivalent (mg/g) using the equation based on the calibration curve: y = 0.025x, R^2 ^= 0.9812, where × is the absorbance and y is the quercetin equivalent (QE).

### Determination of total phenol

The total phenolic content of the extract was determined by the modified Folin-Ciocalteu method [20]. The extract (1 mg/ml) was mixed with 5 ml of Folin-Ciocalteu reagent (previously diluted with distilled water 1:10 v/v) and 4 ml (75 g/l) of sodium carbonate. The mixture was vortexed for 15 s and allowed to stand for 30 min at 40°C for colour development. The absorbance was measured at 765 nm using a spectrophotometer. The content of total phenolic compounds was expressed as mg/g gallic acid equivalent of dry extract sample using the equation obtained from the calibration curve: y = 0.1216x, R^2 ^= 0.9365, where × is the absorbance and y is the tannic acid equivalent (GAE).

### Determination of total proanthocyanidins

The procedure reported by Sun et al. [21] was used in the determination of the total proanthocyanidins. A volume of 0.5 ml of 0.1 mg/ml extract solution was mixed with 3 ml of 4% vanillin-methanol solution and 1.5 ml hydrochloric acid. The mixture was allowed to stand for 15 min while the absorbance was measured at 500 nm. Total proanthocyanidin content was expressed as catechin equivalents (mg/g) using the equation based on the calibration curve: y = 1.8223x + 0.0157, R^2 ^= 0.7246, where × is the absorbance and y is the catechin equivalent (CE).

### Determination of ferric reducing power

A spectrophotometric method [22] was used for the measurement of reducing power. The different concentrations of the extracts and the standards, rutin and BHT (0.02-0.10 mg/ml; 1 ml) were mixed with phosphate buffer (2.5 ml, 0.2 M, pH 6.6) and potassium ferricyanide [K_3_Fe(CN)_6_] (2.5 ml, 1% w/v). The mixture was incubated at 50°C for 20 min. 2.5 ml of trichloroacetic acid (TCA) (10% w/v) was added to the mixture which was centrifuged at 1000 rpm for 10 min. The upper layer of the solution (2.5 ml) was mixed with 2.5 ml distilled water and 0.5 ml of (0.1% w/v) FeCl_3_. While the absorbance was measured at 700 nm in a spectrophotometer, increasing absorbance of the reaction mixture indicated greater reducing power of the samples [23].

### DPPH radical scavenging assay

For DPPH assay, the method of Liyana-Pathirana and Shadidi [24] was adopted. A solution of 0.135 mM DPPH in methanol was prepared and 1 ml of this solution was mixed with 1 ml of different concentrations of the different extracts. The reaction mixture was vortexed thoroughly and left in the dark at room temperature for 30 min. BHT was used as reference standard while methanol was used as control. Reduction of the stable DPPH radical was used as a marker of antioxidant capacity of *Z. mucronata subsp. mucronata *extracts. The changes in colour from deep-violet to light-yellow were measured at 517 nm wavelength using 95% methanolic solution as a reference solution. This was related to the absorbance of the control without the plant extracts. The percentage inhibition of free radical DPPH was calculated from the following equation: % inhibition = [(absorbance of control - absorbance of sample)/absorbance of control] × 100%. All the tests were carried out in triplicates. Though the activity is expressed as 50% inhibitory concentration (IC_50_) [25,26], IC_50 _was calculated based on the percentage of DPPH radicals scavenged. The lower the IC_50 _value, the higher is the antioxidant activity.

### ABTS radical scavenging assay

For ABTS assay, the methods of Johnstone et al., [27] were modified and adopted. The stock solutions included 7 mM ABTS solution and 2.4 mM potassium persulfate solution. The working solution was then prepared by mixing the two stock solutions in equal proportions and allowing them to react for 12 h at room temperature in the dark. The solution was then diluted by mixing 1 ml ABTS^+ ^solution with 60 ml of methanol to obtain an absorbance of 0.708 ± 0.001 units at 734 nm using the spectrophotometer. ABTS^+ ^solution was freshly prepared for each assay. Plant extracts (1 ml) were allowed to react with 1 ml of the ABTS^+ ^solution and the absorbance was taken at 734 nm after 7 min using the spectrophotometer. The ABTS^+ ^scavenging capacity of the extract was compared with that of butylated hydroxy toluene (BHT). The percentage inhibition was calculated as I% = [(A_blank _- A_sample_)/A_blank _] × 100 where A_blank _is the absorbance of ABTS radical + methanol used as control; A_sample _is the absorbance of ABTS radical + sample extract/standard. All the tests were carried out in triplicates. Though the activity is expressed as 50% inhibitory concentration (IC_50_) [25,26], IC_50 _was calculated based on the percentage of ABTS radicals scavenged. The lower the IC_50 _value, the higher is the antioxidant activity.

### Statistical Analysis

Data were expressed as means ± standard deviations (SD) of three replicate determinations and then analyzed by SPSS V.16 (Statistical Program for Social Sciences, SPSS Corporation, Chicago, IL). One way analysis of variance (ANOVA) and the Duncan's New Multiple-range test were used to determine the differences among the means. P values < 0.05 were regarded to be significant. The Pearson correlation analysis was performed between antioxidant activity and total phenolic content.

## Results

### Phytochemical compositions

**Figure 1:**
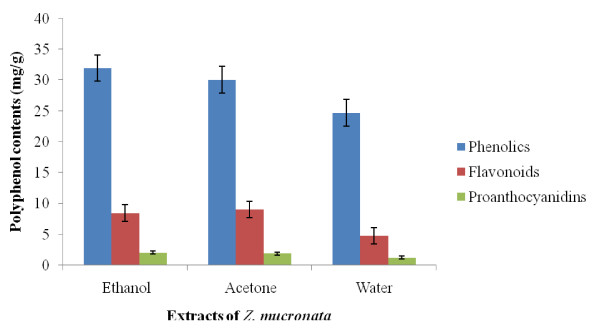
**Polyphenolic contents (mg/g) of *Ziziphus mucronata *subsp. *mucronata***. Data are presented as means ± standard deviation of three replicate with significant increases from all samples tested.

In this study, the result showed that the amount of total phenolic content, flavonoids and proanthocyanidins differ significantly among the various extracts of the *Z. mucronata *subsp*. mucronata *(Figure 1). The values of total phenolic contents varied from 24.72 ± 0.01 to 31.96 ± 0.01 mg GAE/100 g dry weight of plant material. The flavonoid contents values ranged from 4.80 ± 0.01 to 9.02 ± 0.01 mg QE/100 g of dry plant material. The quantity of the proanthocyanidin contents ranged from 1.26 ± 0.01 to 2.08 ± 0.01 mg CE/100 g of dry plant material. The ethanol extract had the highest total phenolic content values, followed by the acetone extract while water extract contained the least. The highest value of flavonoid was recorded in the acetone extract, followed by the ethanol extract and the water extract. Though proanthocyanidin was more in ethanol extract than other extracts, the proanthocyanidins content in this plant was relatively low and the differences in its quantity from one extract to another are significant. These results showed that, quantitatively, in *Z. mucronata*, total phenol contents were significantly higher than the flavonoid contents while the proanthocyanidins were the least.

### Ferric Reducing Antioxidant Power (FRAP)

**Figure 2:**
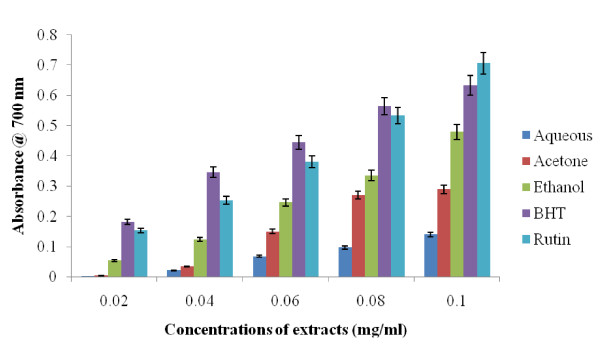
**Ferric reducing power determinations for the alcoholic and aqueous extracts of *Z. mucronata *subsp*. mucronata***. Data are presented as means ± standard deviation of three replicate with significant increases from all samples tested.

The FRAP values of different extracts of *Z. mucronata *subsp*. mucronata *was determined in an attempt to compare their antioxidant activities The reducing abilities of the different extracts determined by FRAP method were measured spectrophotometrically by their absorbances at 700 nm and summarized in Figure 2. The reducing ability of the extracts showed a dose-dependent trend increasing with increases in the concentrations of the extracts. While highest reducing ability was observed at the highest concentration of each of the extracts, significant differences existed between the reducing ability of each all the extracts. Of the three extracts, acetone extract exhibited the highest reducing capability (0.454 ± 0.001) at the highest concentration. This was followed by (0.421 ± 0.002) ethanol extract while aqueous extract (0.14 ± 0.002) was the least. The reducing abilities recorded was in the following order, Rutin > BHT > Acetone > Ethanol > Aqueous which showed that rutin exhibited the highest reducing ability at 0.1 mg/ml. The significant differences for free radical scavenging activity among the different extracts may be attributed to the varied quantity of each of the phytochemical contents of the plant.

### DPPH radical scavenging activity

The results of the DPPH scavenging activity of the extracts are as shown in Table 1. The alcoholic and aqueous extracts of *Z. mucronata *stem bark exhibited concentration dependent antiradical activity by inhibiting DPPH radical with inhibitory concentration 50% (IC_50_) values of 0.0646 ± 0.02 mg/ml (aqueous), 0.0482 ± 0.02 mg/ml (acetone) and 0.0422 ± 0.03 mg/ml (ethanol) while those of the standards were 0.0406 ± 0.02 mg/ml (BHT) and 0.0411 ± 0.02 mg/ml (vitamin C). This result agreed with the earlier report of Motalleb et al. [28] which showed that the scavenging effects on the DPPH radical increases sharply with the increasing concentration of the samples and standards to a certain extent. In the order of activity, ethanol had the stronger antioxidant activity (0.0422 ± 0.03 mg/ml), followed by acetone extract while the least activity was obtained from aqueous extract. However, all the extracts exhibited significant DPPH free radical scavenging activity comparable to those obtained from the standards used as controls.

**Table 1:** DPPH radical scavenging activity of the alcoholic and aqueous extracts of *Z. mucronata *subsp. *mucronata*

	% inhibitions of ABTS^+ ^± SD at different concentrations of the extracts					
**Extracts**	**0.02 (mg/ml)**	**0.04 (mg/ml)**	**0.06 (mg/ml)**	**0.08 (mg/ml)**	**0.1 (mg/ml)**	**IC_50 _(mg/ml)**

Aqueous	9.87 ± 0.02^a^	21.86 ± 0.02^b^	46.26 ± 0.02^c^	62.18 ± 0.02^d^	70.34 ± 0.02^e^	0.0646 ± 0.02
Acetone	32.67 ± 0.02^a^	39.35 ± 0.03^b^	64.49 ± 0.02^c^	82.53 ± 0.03^d^	88.15 ± 0.02^e^	0.0482 ± 0.02
Ethanol	34.35 ± 0.02^a^	47.55 ± 0.03^b^	71.04 ± 0.02^c^	85.06 ± 0.01^d^	94.47 ± 0.02^e^	0.0422 ± 0.03
BHT	37.36 ± 0.02^a^	48.50 ± 0.03^b^	74.52 ± 0.01^c^	92.56 ± 0.03^d^	98.47 ± 0.02^e^	0.0406 ± 0.02
Vitamin C	23.88 ± 0.01^a^	47.65 ± 0.02^b^	73.89 ± 0.03^c^	90.94 ± 0.01^d^	93.01 ± 0.03^e^	0.0411 ± 0.02

Data are presented as means ± standard deviation of three replicate with significant increases from all samples tested.

Superscript a, b, c, d and e showed that means ± standard deviation in the same row with different superscript are significantly different (p < 0.05)

### ABTS radical scavenging activity

Significant ABTS^+ ^free radical scavenging activity was evident in both alcoholic and aqueous extracts (Table 2). The rate of the decrease in absorbance at 734 nm depends on the type and amount of antioxidants and the results are thus expressed as antioxidant standard equivalents [29,30]. In this study, the suppression of the absorbance of ABTS^+ ^in a concentration-dependent manner is typically shown by all the extracts. Acetone (97.71 ± 0.03) and ethanol (95.73 ± 0.02) extracts showed ABTS^+ ^radical-scavenging activity higher than the reference standard BHT (93.47 ± 0.03) and aqueous extracts (88.96 ± 0.02) was lower than that of the BHT. Ethanol extract showed a 50% inhibitory concentration of IC_50 _= 0.0306 ± 0.04 mg/ml while acetone extract showed IC_50 _= 0.0317 ± 0.04 mg/ml which are higher than IC_50 _= 0.0429 ± 0.04 mg/ml of the aqueous extract. The differences in the antioxidant activities of the three extracts are statistically significant (p < 0.05).

**Table 2:** ABTS radical scavenging activity of the alcoholic and aqueous extracts of *Z. mucronata *subsp. *mucronata*

	% inhibitions of ABTS^+ ^± SD at different concentrations of the extracts					
**Extracts**	**0.02 (mg/ml)**	**0.04 (mg/ml)**	**0.06 (mg/ml)**	**0.08 (mg/ml)**	**0.1 (mg/ml)**	**IC_50 _(mg/ml)**

Aqueous	23.95 ± 0.02^a^	43.49 ± 0.06^b^	72.96 ± 0.02^c^	82.05 ± 0.03^d^	88.96 ± 0.02^e^	0.0429 ± 0.04
Acetone	42.13 ± 0.05^a^	52.47 ± 0.03^b^	75.64 ± 0.04^c^	92.48 ± 0.04^d^	97.71 ± 0.03^e^	0.0317 ± 0.04
Ethanol	43.10 ± 0.03^a^	54.85 ± 0.04^b^	82.76 ± 0.04^c^	93.76 ± 0.02^d^	95.73 ± 0.02^e^	0.0306 ± 0.04
BHT	33.31 ± 0.02^a^	54.27 ± 0.02^b^	79.06 ± 0.02^c^	91.05 ± 0.05^d^	93.47 ± 0.03^e^	0.0343 ± 0.02

Data are presented as means ± standard deviation of three replicate with significant increases from all samples tested. Superscript a, b, c, d and e showed that means ± standard deviation in the same row with different superscript are significantly different (p < 0.05)

## Discussions

### Phytochemical compositions

Phenolic compounds and flavonoids are the major constituents in most plants reported to possess antioxidant and free radical scavenging activity [31,32]. Phenolic compounds are efficient free radical scavengers [33] exhibiting antioxidant activity by inactivating lipid free radicals, or by preventing the decomposition of hydroperoxides into free radicals [34]. Medicinal plants containing flavonoids exhibit multiple pharmacological activities [35]. Flavonoids, probably the largest of the natural phenolics [36], possess antioxidant properties [37] acting as effective scavenger of deleterious free radicals and reactive oxygen species [38,39]. They have several biological activities including anti-inflammatory, antiallergic and immunomodulatory properties which could help to prevent age-related cardiovascular and neurodegenerative diseases as well as reducing the risk of cancer [40]. Their antioxidant activity depends on the number and location of hydroxyl groups of the flavonoid ring system and the relationship between peroxyl radical absorbing activity and the number of hydroxyl groups in flavonoids [41]. While they act through scavenging or chelating process [42], their ability to stabilize membranes by decreasing the fluidity of the membranes as well as partitioning flavonoids into the hydrophobic core of the membrane [43] have been considered a contributory mechanism to their antioxidant activities. Since antioxidants are used for the prevention and treatment of free radical-related disorders [44] as well as being essential in the prevention of diseases [5], the high quantity of phenolic and flavonoid contents of *Z. mucronata *subsp*. mucronata *may contribute to its potential antioxidant property and curative ability adsorbing and neutralizing free radicals.

### Ferric Reducing Antioxidant Power (FRAP)

The best known antioxidants are phenolic compounds and flavonoids [45-47] exhibiting extensive free radical scavenging activities through their reactivity as hydrogen or electron-donating agents and metal ion chelating properties [7]. In this study, the reductive ability of the extracts reflected the reducing power of the *Z. mucronata *as a potential source of antioxidants. The FRAP assay showed that the antioxidants contained in the extracts acted as reductants in a redox-linked colorimetric reaction [32]. This report agreed with several reports that showed a close relationship between total phenolic content and high antioxidant activity [48-50]. This study, therefore, suggests that the recorded antioxidant capacity resulted from the contribution of different phytochemicals present in the plant and the reducing capacity of each extract may serve as a significant indicator of the potential activity of *Z. mucronata*.

### DPPH radical scavenging activity

The reaction of DPPH with numerous antioxidants has earlier been published and the stoichiometry characterized [51]. The DPPH antioxidant assay is based on the principle that 2,2-diphenyl-1-picryl-hydrazyl (DPPH) is able to decolourise in the presence of free radical scavengers (antioxidants). The color turns from purple to yellow as the molar absorptivity of the DPPH radical at 517 nm reduces from 9660 to 1640 when the odd electron of DPPH radical becomes paired with hydrogen from a free radical scavenging antioxidant to form the reduced DPPH-H. The odd electron in the DPPH radical is responsible for the absorbance at 517 nm and also for the visible deep purple colour [52]. Antioxidants in the different extracts of *Z. mucronata subsp. mucronata *reacted with DPPH which is reduced to the DPPH-H. Consequently, the absorbencies decreased from the DPPH radical to the DPPH-H formed. The degree of discoloration indicated the scavenging potential of the extracts in terms of hydrogen donating ability. The scavenging ability of this plant was significant and corresponded to the presence of high quantity of phenolic compounds. It is, therefore, reasonable to conclude that high antioxidants in the different extracts of *Z. mucronata *subsp. *mucronata *have resulted in the high level of DPPH radical scavenged in this study.

### ABTS radical scavenging activity

The fact that phenolic antioxidants have been reported to scavenge ABTS^+ ^through hydrogen atom donation [53], electron transfer or even with a combination of the two mechanisms [54] may explain a current interest in the applicability of the ABTS^+ ^assay in determining the radical scavenging activities of plant extracts. Like the DPPH assay, ABTS assay measured the total antioxidant activity of the extracts. The different antioxidant activities of the different extracts and the ability of ethanolic extract to exhibit radical-scavenging greater than other extracts may indicate that active compounds of different polarity could be present in *Z. mucronata*. While the hydroxyl (OH) group in aromatic ring have been related to extracts' activity towards ABTS [54], the significant ABTS^+ ^scavenging activity of the different extracts may, however, depend on the presence of higher number of hydroxyl groups present in the plant while the high antioxidant activities may be due to their flavonoids and phenolic contents.

## Conclusion

Today, antioxidative properties of extracts from plants have become a great interest due to their possible uses as natural additives to replace synthetic ones. This study was designed to investigate the phenolic contents and evaluate the *in vitro *antioxidant activities of *Z. mucronata *subsp. *mucronata*. Phenolic compounds, flavonoids and proanthocyanidins were detected in the various extracts of the plant. The antioxidant potential of the extracts indicated that the alcoholic extracts exhibited higher antioxidant activities than the aqueous extracts. The results of FRAP, DPPH and ABTS assays showed that the extracts possess not only the antioxidant activities, but also potent free radical scavenger capability. The antioxidant activity of the extracts correlated well with the total phenolic contents and indicated that phenolic compounds are dominant contributors to the antioxidant activity of the extracts. The high polyphenolic contents, especially flavonoids, responsible for antioxidant activity may be the mechanism of action. *Z. mucronata *subsp. *mucronata *is a potential free radical scavenger and a useful source of natural antioxidants which may justify the therapeutic effectiveness of the plant.

## Competing interests

The authors declare that they have no competing interests.

## Authors' contributions

OOO participated in the design of the study, prepared the extracts, carried out the study and drafted the manuscript. AJA coordinated the study and revised the manuscript. Both authors approved the final manuscript.

## Pre-publication history

The pre-publication history for this paper can be accessed here:

http://www.biomedcentral.com/1472-6882/11/130/prepub
